# Equipping our public health nutrition workforce to promote planetary health: a case example of tertiary education co-designed with students

**DOI:** 10.1017/S1368980024002611

**Published:** 2025-01-07

**Authors:** Ka Po Chau, Wing Chong, Raquel Londono, Beau Cubillo, Julia McCartan, Liza Barbour

**Affiliations:** 1 Department of Nutrition, Dietetics and Food, Monash University, Melbourne 3168, Australia; 2 Menzies School of Health Research, Charles Darwin University, Darwin, Australia; 3 Monash Centre for Scholarship in Health Education (MCSHE), Monash University, Melbourne, Australia

**Keywords:** Planetary health, Co-design, Capacity building, Tertiary education

## Abstract

**Objective:**

The public health nutrition workforce is well placed to contribute to bold climate action; however, tertiary educators are seeking practical examples of how to adequately prepare our future workforce. This study examines the responses of university students engaged in a co-designed planetary health education workshop as part of their public health nutrition training.

**Design:**

A mixed-methods approach was used to collect and interpret student responses to four interactive tasks facilitated during an in-person workshop. Data were analysed using statistical tests, frequency counting and content analysis.

**Setting:**

The intervention was co-designed by students (*n* 5) and an educator over a 4-week period as part of a larger multi-disciplinary study at an Australian university.

**Participants:**

The workshop engaged nutrition and dietetics students (*n* 44) enrolled in public health nutrition coursework.

**Results:**

Students reported an increase in self-perceived knowledge about planetary health as a concept and how they can promote it within their future professional roles. Students’ descriptions of what planetary health means to them were focused on humans’ role in protecting and preserving the ecosystem, the responsible and sustainable use of natural resources and a need to sustain a healthy life for future generations. Students prioritised the values of ‘collaboration’ and ‘respect’ as being critical to guide personal and professional practice to promote planetary health.

**Conclusions:**

This study demonstrated that incorporating planetary health curricula designed by, and for, university students could be a feasible and effective way to prepare the future public health nutrition workforce to address planetary health challenges.

Planetary health is an emerging field that focusses on ‘analysing and addressing the impacts of human disruptions to Earth’s natural systems on human health and all life on earth’^(1)^. The public health nutrition workforce has the capacity to promote planetary health in a diverse range of settings and population groups; therefore, it plays a critical role in addressing the triple planetary health crises of climate change, pollution and biodiversity loss^(2–4)^. However, the window for transformative action is rapidly closing^(2,5)^. Our future health workforce will be at the coalface required to address these major public health challenges. However, they are not receiving sufficient fit-for-purpose education during their tertiary public health nutrition training to prepare them adequately^(6,7)^. Evidence suggests that educators agree this should be part of health professions’ training; however, they themselves do not feel prepared to facilitate planetary health education^(7–9)^.

Research has explored the degree to which various health disciplines are embedding planetary health education in their training, and theoretically how this is being done^(10–12)^, including public health^(13)^ and nutrition and dietetics^(14–16)^. However, to our knowledge, the peer-reviewed literature is lacking practical examples of planetary health education interventions amongst public health nutrition learners that include an embedded evaluation to assess efficacy. This research examines the responses of nutrition and dietetics students engaged in a planetary health education workshop, co-designed by students and their educator, as part of their tertiary public health nutrition training.

## Methods

### Setting and population

A 3-hour planetary health workshop was facilitated face to face on campus at an Australian university with students enrolled in a Public Health Nutrition module as part of their Bachelor of Nutrition Science and Master of Nutrition and Dietetics degrees. The lesson plan and teaching materials (Table 1: Workshop learning objectives and outline) were co-designed by students (*n* 5) and educators (*n* 1). The authors of this paper were all involved in this process including three students (shared first-authorship).

**Table 1.** Workshop learning objectives and outline co-designed with students

Learning objectives	By the end of this session, students will be able to: • Understand our role and responsibility as health professionals in promoting planetary health. • Engage in self-reflection about your own values and practices and how they impact others. • Feel equipped and empowered to become a planetary health ambassador.
Workshop outline	Pre-class activities • A short video introducing planetary health, a case study (news article and video) of the impact of climate-related events on public health and a self-reflective activity about core values. In-class activities • Pre-poll followed by word cloud activity • Overview of planetary health theory including Indigenous perspectives and reflective activity on alarming statistics • Guided activity to first reflect on pre-class activity about personal core values, followed by a group task to apply these to five workplace scenarios Post-class activities • Prompt to read the Planetary Health Pledge and reflect on how this might impact future practice as a health professional.

### Study design

This study is part of a Faculty-wide intervention to increase the quantity and quality of planetary health curricula offered within health professions’ degrees at the University. A co-design approach was adopted with educators and students from twelve disciplines^(17)^, which will be examined and published elsewhere.

This research utilised both qualitative and quantitative methods to examine (i) students’ attitudes, self-efficacy and self-perceived knowledge regarding planetary health, (ii) students’ word association with the term ‘planetary health’, (iii) students’ self-perception of the term ‘planetary health’ and (iv) students’ prioritisation of core values to apply in practice. This study was approved by the Monash University Human Research Ethics Committee (ID: 38428)

### Data collection and analysis

Data were collected from students through interactive tasks during the facilitated workshop and align with the four study aims outlined previously.

#### Students’ attitudes, self-efficacy and self-perceived knowledge regarding planetary health

Workshop attendance was self-recorded by students using Moodle, an e-learning platform. Students were invited to complete both a pre- and post-workshop poll, containing 5 identical questions. These questions were based on a previously administered survey exploring healthcare professionals’ attitudes, self-efficacy and self-perceived knowledge regarding planetary health^(7)^. All poll questions prompted a Likert scale response, with a four-point rating allocated for analysis purposes from strongly disagree, disagree, agree and strongly agree. These quantitative results were analysed using an online statistical software GraphPad, whereby an independent sample t-test was performed to compare pre- and post-poll data, as data were collected anonymously so students were not matched.

#### Students’ word association with the term ‘planetary health’

In addition to the pre-post polls, students were encouraged to participate in a number of interactive tasks throughout the workshop. To seek the students’ immediate associations, they were asked ‘What words come to mind when you think about “Planetary Health”?’ at the beginning of the workshop using an online polling software. A word cloud was generated using student responses, illustrating the frequency of words, with the size of each word corresponding to how frequently that term appeared in the analysed text^(18)^. The utilisation of word clouds is growing in academic and educational settings due to their capacity to stimulate critical thinking and student engagement^(19)^.

#### Students’ perception of the term ‘planetary health’

The question: ‘What *does planetary health mean to you?’* was also asked at the beginning of the workshop and answers were recorded using online polling software. Content analysis was used to interpret the poll responses. Content analysis enables the reduction of broad descriptions to refined concepts that are situated within the context of the overarching research question^(20,21)^. The content analysis procedure used for this study was adapted from several papers^(21–23)^ and involved transferring the poll results to a spreadsheet, identifying keywords within the responses (code words), creating subcategories by grouping similar code words together and finally grouping these subcategories into main concepts. This process was implemented by three co-authors (BC, LB and RL) who worked together to consider decisions regarding categorisation and concept descriptions collaboratively.

#### Students’ prioritisation of core values to apply in practice

The main workshop activity involved the presentation of five scenarios of typical work roles for practicing nutritionists and dietitians. This activity encouraged students to reflect on the personal qualities and values required in their future roles as nutrition professionals. Prior to the activity, the students reviewed a list of values created by Brene Brown^(24)^, along with a visual prompt that included three example values considered fundamental to the promotion of planetary health. Students then worked in groups to write down the values they felt were associated with each of the five scenarios and consider how these values relate to planetary health. To encourage dialogue, a ‘world cafe’ approach was utilised as a participatory method of data collection from the large group of student participants^(25)^. This involved each student group rotating between all five scenarios and contributing to a collective butcher’s paper list of relevant values. Frequency analysis was conducted to review which core values students prioritised.

## Results

Research findings are presented here, to address each of the four key study aims.

### Students’ attitudes, self-efficacy and self-perceived knowledge regarding planetary health

Of all students in attendance at the workshop (*n* 44), 40 participants completed the pre-workshop poll (response rate 90·9 %) and 30 completed the post-workshop poll (response rate 68·2 %). Poll results demonstrated statistically significant improvements in four of the five questions (see online supplementary material, Supplemental Material 1: Pre- and Post-workshop Poll Results). Students reported an increased ability to explain the term ‘planetary health’ (*P*< 0·0001), describe how health professionals in their field can address the causes and consequences of climate change (*P*= 0·001), advocate for change to promote planetary health within their field (*P*< 0·0001) and believe their training had prepared them to address the causes and consequences of climate change (*P*= 0·0001). Students reported an increase in agreement that planetary health is core business for healthcare professionals; however; this result was not statistically significant when compared before and after the workshop (*P*= 0·0668).

### Students’ word association with the term ‘planetary health’

**Figure 1.**
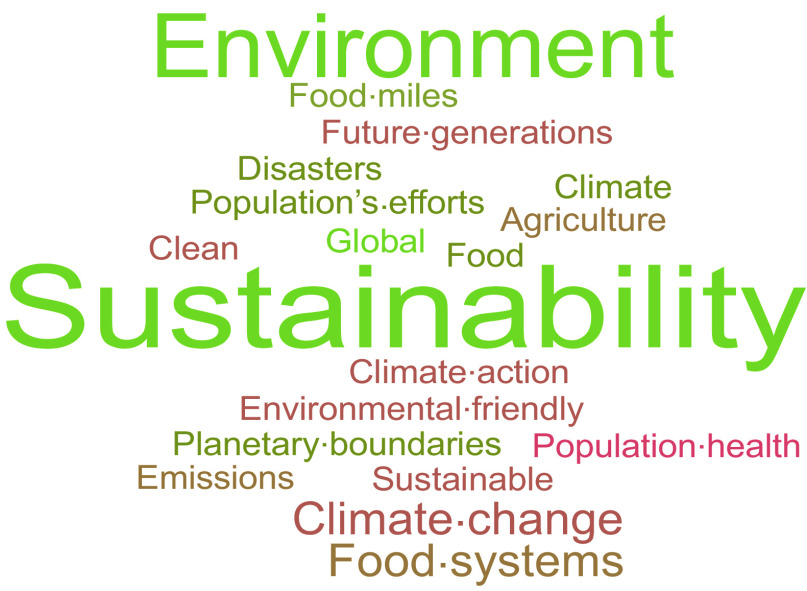
Word Cloud of responses to ‘What words come to mind when you think about “Planetary Health”?’.

Among the responses (*n* 29) (Figure 1: Word Cloud of responses to ‘What words come to mind when you think about “Planetary Health”?’), the term ‘*sustainability*’ was the most prevailing within the word cloud (*n* 9), followed by ‘*environment*’ (*n* 5). Other frequently submitted words were ‘*climate change*’, ‘*climate action*’ and ‘*environmentally friendly*’.

### Students’ perception of the term ‘planetary health’

**Figure 2.**
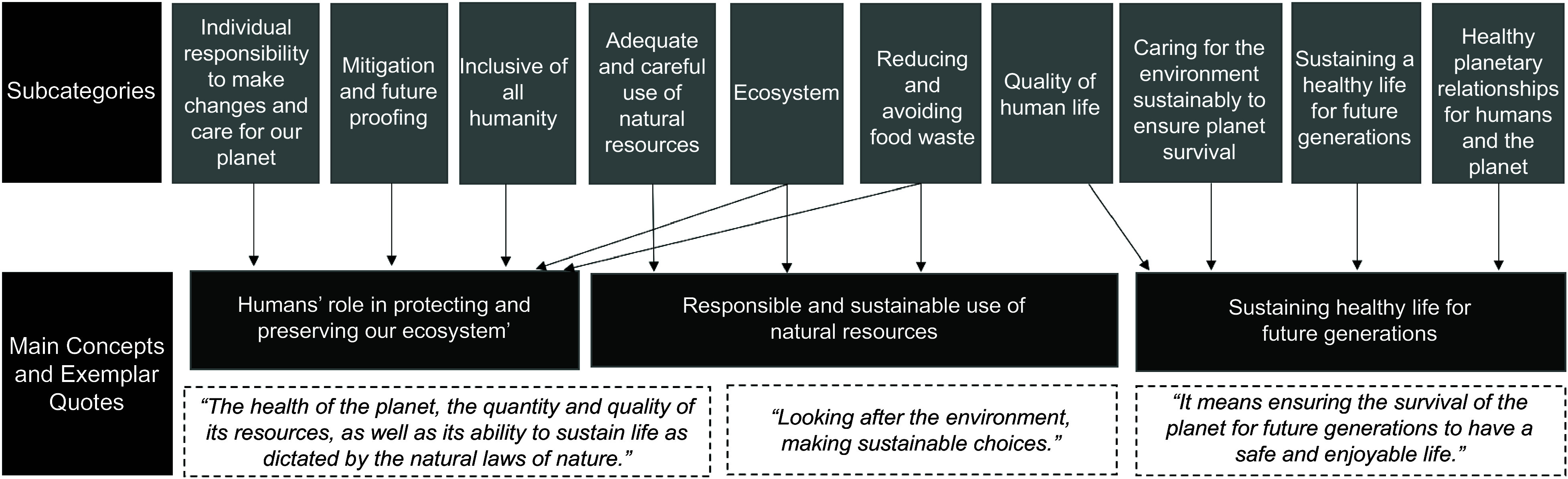
Content analysis of responses ‘What does planetary health mean to you?’.

Content analysis of the responses (*n* 29) (Figure 2: Responses to ‘What does planetary health mean to you?’) resulted in 10 subcategories derived from the de-contextualisation of several codes developed from passages of text as the unit of analysis (see online supplementary material, Supplemental Material 2: Content analysis of responses to: What does planetary health mean to you?). The subcategories were then abstracted into higher order and classified into three main concepts as a means to re-contextualise and interpret the data. Exemplar quotes display the exact wording from students to illustrate the inferences drawn from the main concepts.

### Students’ prioritisation of core values to apply in practice

The world café activity yielded a total of 123 values documented by students across the five scenarios (see online supplementary material, Supplemental Material 3: Frequency analysis of ‘Value’). Of the ten butchers’ paper, with two assigned to each scenario, students documented ‘*collaboration*’ on six, making it the most frequently written value. This finding aligns with workshop content delivered prior to this activity (see online supplementary material, Supplemental Material 4: Visual prompt prior to World Café activity) that highlighted the need for interdisciplinary collaboration to address emerging planetary health crises^(26,27)^. ‘*Respect*’ was identified as a core quality required by nutritionists and dietitians within all five of the scenarios. This observation also aligns with workshop content that emphasised the critical role of respecting planetary boundaries and Indigenous knowledge, perspectives and values systems in addressing planetary health challenges. While ‘*sustainability*’ and ‘*environment*’ are recognised in the literature as crucial values to addressing planetary health issues^(28)^, these only appeared once on the butchers’ paper, suggesting a lower priority.

## Discussion

This study demonstrated that a co-designed in-person workshop can effectively increase self-perceived knowledge and self-efficacy scores regarding planetary health action amongst nutrition and dietetics tertiary students studying public health nutrition. Students reported higher levels of self-perceived knowledge about planetary health as a concept and taking action to promote planetary health within their future professional roles. Interestingly, students’ descriptions of what planetary health means to them were focused on humans’ role in protecting and preserving the ecosystem, the responsible and sustainable use of natural resources and a need to sustain a healthy life for future generations, all of which are reflected in Guzman et al.’s (2021) best practice framework for facilitating planetary health education^(29)^. Students prioritised the values of *collaboration* and *respect*, as being critical for nutritionists and dietitians to draw upon in their personal and professional practice to promote planetary health. Both values are broadly referenced as being fundamental to transformative action in the health sector and beyond to achieve sustainable development targets and the desired planetary health outcomes^(3,29)^. Further exploration of students’ perceptions about why certain personal and professional values are critical in applying planetary health principles in practice, and how these values can be nurtured amongst the current and future workforce, is needed.

The learning and teaching activities developed by, and for, public health nutrition learners are described in this study, together with an analysis of student responses to each task. These learning and teaching activities can be used to assist health professions educators as they strive to embed more planetary health education in their curricula. There is broad agreement that this curriculum shift must happen^(6,29–31)^, and evidence that educators are seeking practical examples of what this looks like in practice for the various health disciplines including public health^(16)^. While some case examples of tertiary-level sustainable food systems education in the public health nutrition field are available^(32)^, to our knowledge no studies have published their efficacy amongst public health nutrition learners. It is a limitation that pre-post poll data is not matched and that the post-poll data reflects a lower response rate (68 %), likely missing students who were less engaged with the workshop material which would have impacted the statistical significance of this data. Another limitation of this present study is that it is a small intervention involving one single discipline, it offers a unique evaluation of curricula that has be co-designed with, and for, public health nutrition students on the emerging topic of planetary health.

### Conclusion

It is well established that public health outcomes are dependent on the health of the natural living systems that humans are part of. However, those responsible for training the future public health workforce to address contemporary food-related environmental challenges have limited capacity and guidance to offer effective, evidence-informed planetary health education. More practical case studies of how planetary health education can be facilitated in the tertiary setting are required, particularly those that have been evaluated. This study demonstrated that an interactive workshop for public health nutrition students can effectively increase self-perceived knowledge about what planetary health is, how our future workforce can address the causes and consequences of climate change in practice and how they can advocate for change to promote planetary health. Our results show that incorporating planetary health curricula designed by, and for, tertiary learners could be a feasible and effective way to prepare future healthcare professionals to face planetary health challenges.

## Supporting information

Chau et al. supplementary material Chau et al. supplementary material

## Supplementary material

For supplementary material accompanying this paper visit https://doi.org/10.1017/S1368980024002611
