# Supplementary material for: Equipping our public health nutrition workforce to promote planetary health: a case example of tertiary education co-designed with students
Source: Public Health Nutr. 2025 Jan 7;28(1):e29. doi: 10.1017/S1368980024002611 (PMC11822643; doi:10.1017/S1368980024002611)
Supplement: Chau et al. supplementary material [file S1368980024002611sup001.docx]

**Supplemental Material 1** Pre- and Post-workshop Poll Results *(Independent sample t-test)*

|  |  | Q1 | Q2 | Q3 | Q4 | Q5 |
| --- | --- | --- | --- | --- | --- | --- |
|  |  | I know how best to explain what planetary health means for healthcare professionals. | I know what healthcare professionals in my field can do to address the causes and consequences of climate change. | Planetary health should be core business for healthcare professionals. | I feel equipped to advocate for change to promote planetary health within my field. | My training has prepared me to address the causes and consequences of climate change within my field. |
| Pre-workshop Poll (n=44) |  |  |  |  |  |  |
|  | Mean | 2.73 | 2.90 | 3.25 | 2.75 | 2.83 |
|  | SD | 0.55 | 0.59 | 0.54 | 0.59 | 0.55 |
|  | SEM | 0.09 | 0.09 | 0.09 | 0.09 | 0.09 |
|  | Response rate % (n) | 90.90 (40.00) | 90.90 (40.00) | 90.90 (40.00) | 90.90 (40.00) | 90.90 (40.00) |
| Post-workshop Poll (n=44) |  |  |  |  |  |  |
|  | Mean | 3.40 | 3.40 | 3.50 | 3.37 | 3.37 |
|  | SD | 0.56 | 0.62 | 0.57 | 0.61 | 0.56 |
|  | SEM | 0.10 | 0.11 | 0.10 | 0.11 | 0.10 |
|  | Response rate % (n) | 68.20 (30.00) | 68.20 (30.00) | 68.20 (30.00) | 68.20 (30.00) | 68.20 (30.00) |
| standard error of difference | | 0.14 | 0.15 | 0.14 | 0.15 | 0.13 |
| df |  | 68.00 | 68.00 | 68.00 | 68.00 | 68.00 |
| t-value |  | 5.01 | 3.43 | 1.86 | 4.26 | 4.06 |
| 95% CI | | [-0.94, -0.41] | [-0.79, -0.21] | [-0.52, 0.02] | [-0.91, -0.33] | [-0.81, -0.28] |
| Mean of pre-poll minus post-poll | | -0.67 | -0.50 | -0.25 | -0.62 | -0.54 |
| p-value |  | <0.0001* | 0.0010* | 0.0668 | <0.0001* | 0.0001* |

Values were rounded to two decimal places, except p-value. Pre- and Post-poll data were based on a scale from 1 to 4; 1 represents strongly disagree and 4 represents strongly agree. Independent sample t-test was performed to assess differences between pre- and post-workshop poll data.

Note: SD = standard deviation; SEM = standard error of mean; Q = question.

* indicate statistical significance.

**Supplemental Material 2** Results from the content analysis of responses to: What does planetary health mean to you?


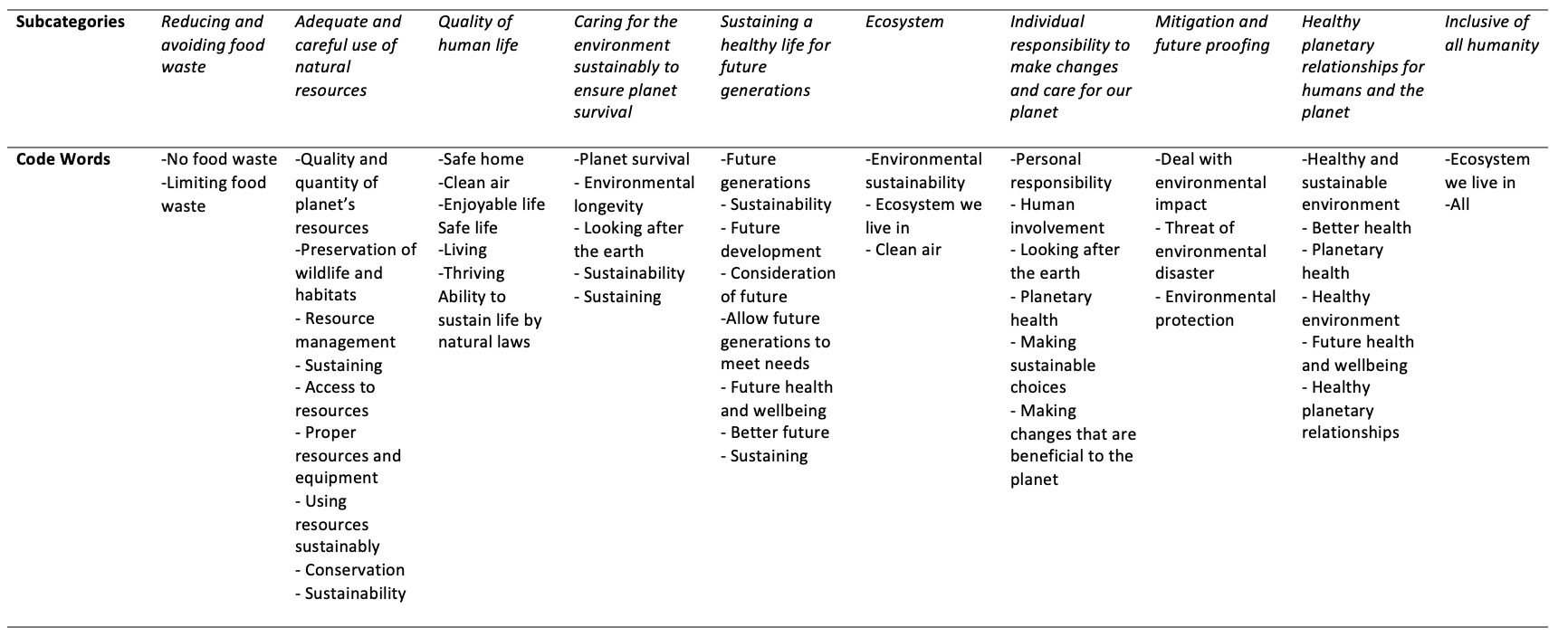


**Supplemental Material 3:** Frequency analysis of “Value” inputs in response to five different nutrition professions scenarios **
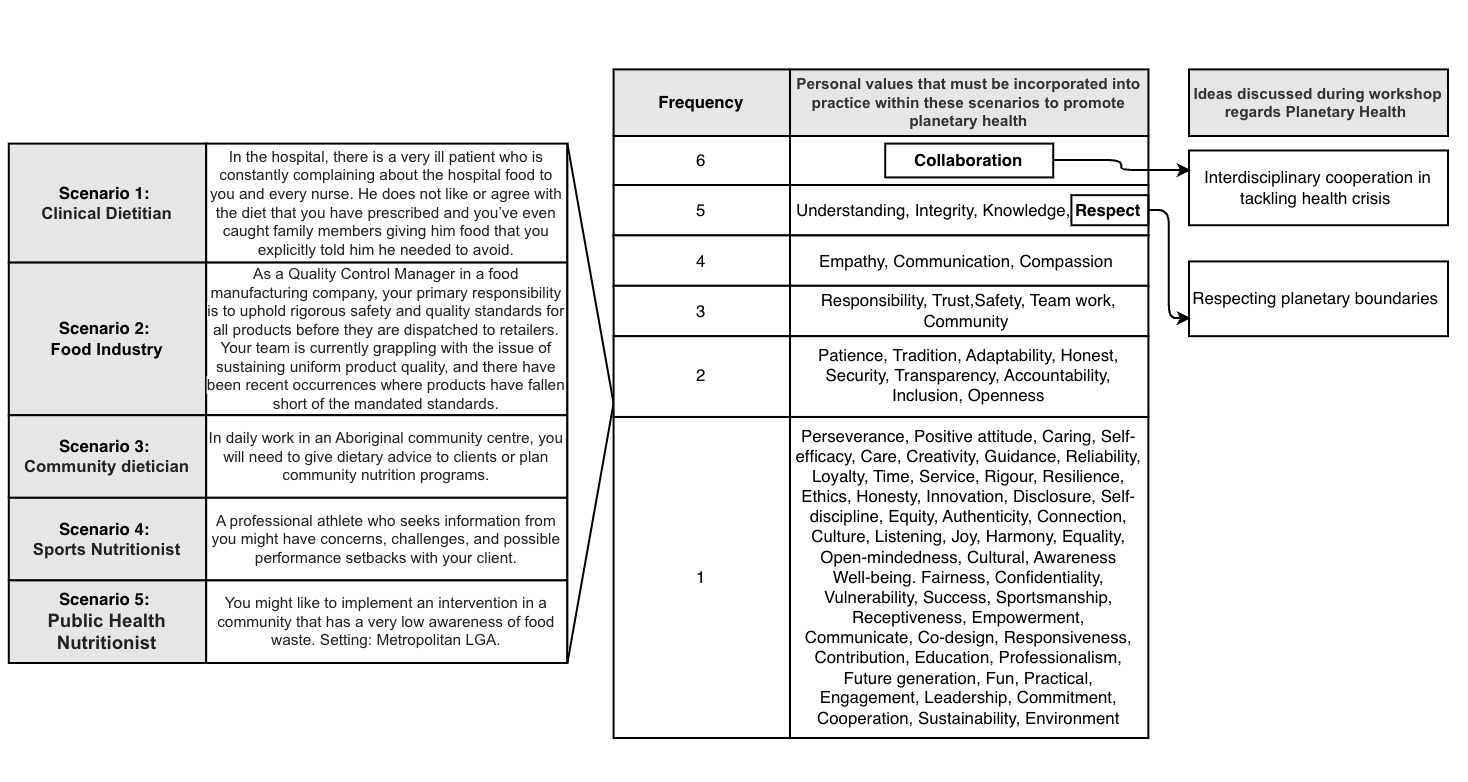
**

**Supplemental Material 4:** Visual prompt for students to review prior World cafe activity

**
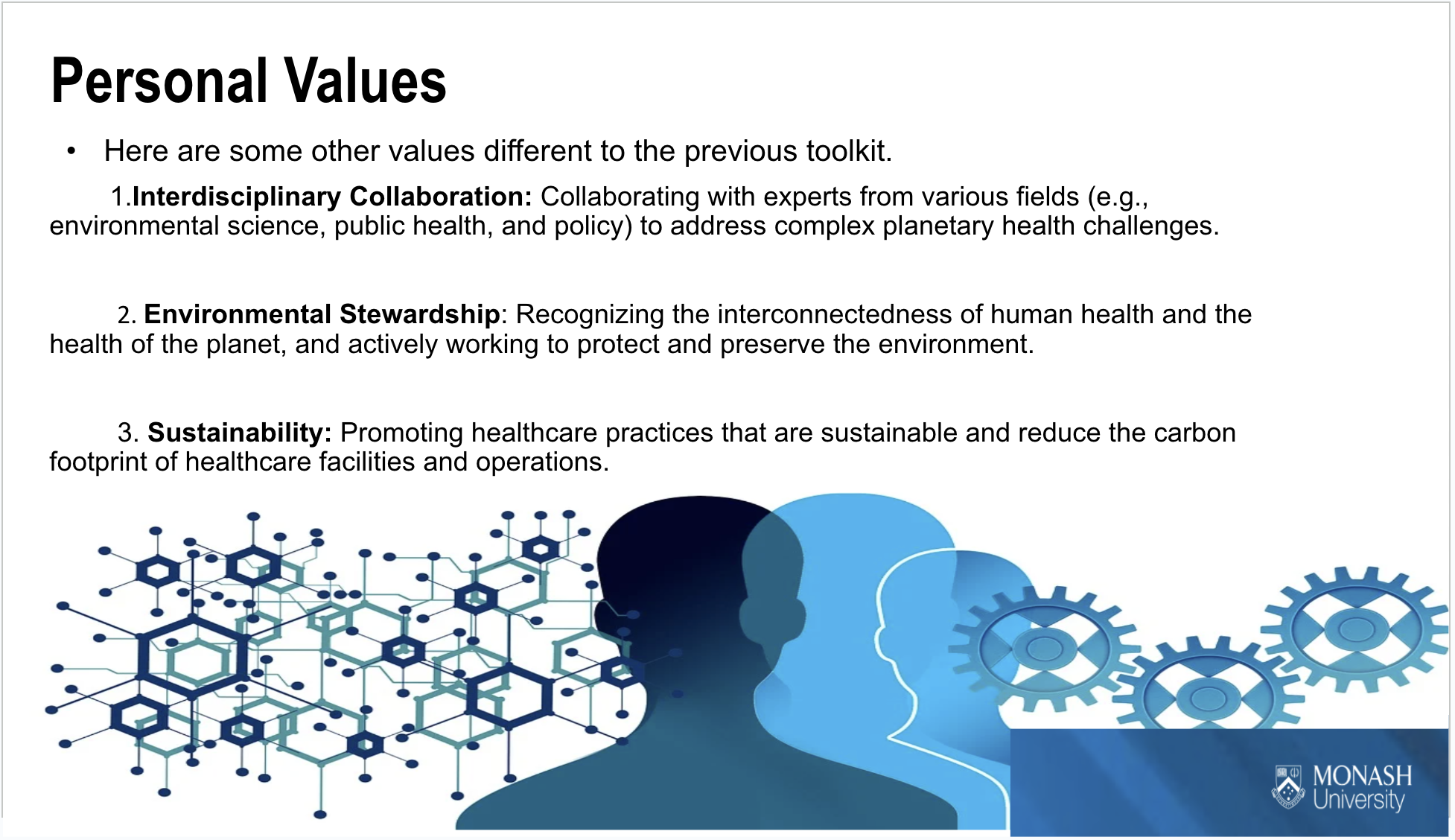
**
